# Innate Recognition of Fungal Cell Walls

**DOI:** 10.1371/journal.ppat.1000758

**Published:** 2010-04-22

**Authors:** Stuart M. Levitz

**Affiliations:** University of Massachusetts Medical School, Worcester, Massachusetts, United States of America; University of California San Francisco, United States of America

The emergence of fungal infections as major causes of morbidity and mortality in immunosuppressed individuals has prompted studies into how the host recognizes fungal pathogens. Fungi are eukaryotes and as such share many similarities with mammalian cells. The most striking difference, though, is the presence of a cell wall that serves to protect the fungus from environmental stresses, particularly osmotic changes [1]. This task is made challenging because the fungus must remodel itself to allow for cell growth and division, including the conversion to different morphotypes, such as occurs during germination of spherical spores into filamentous hyphae. The cell wall also connects the fungus with its environment by triggering intracellular signaling pathways and mediating adhesion to other cells and extracellular matrices. Here, important facts and concepts critical for understanding innate sensing of the fungal cell wall by mammalian pathogens are reviewed.

## There Are Intra- and Interspecies Similarities and Differences in Fungal Cell Wall Composition

The fungal cell wall is predominantly composed of carbohydrate polymers interspersed with glycoproteins. The three major components, found in all medically important fungi studied to date, are β-glucans (polymers of glucose), chitin (polymer of N-acetylglucosamine), and mannans. While these three components are intermingled throughout the cell wall, chitin tends to predominate near the plasma membrane, whereas the mannans have a propensity for the outer cell wall [1], [2]. β-1,3-glucans form the main structural scaffold of the cell wall and have varying amounts of β-1,6 branches. Chitin is thought to mostly add structural strength to the cell wall. Mannans are chains of up to several hundred mannoses that are added to fungal proteins via N- or O-linkages [3]. Mannoproteins can covalently attach to glucans or chitin via either their sugar residues or via glycosylphosphatidylinositol (GPI) links. GPI anchors may also attach mannoproteins to the plasma membrane. Finally, proteins that are normally found in intracellular compartments, such as heat shock proteins, have been found cross-linked in the cell wall in a manner allowing interaction with immune cells [1], [4], [5].

While the above provides a general overview of the cell wall, it is important to emphasize that extensive differences may be found when comparing different fungal species and even when comparing strains within a species. Examples include α-glucans in addition to β-glucans in some (but not all) strains of *Histoplasma capsulatum*, chitosan in *Cryptococcus neoformans*, and galactomannans in species of *Aspergillus* [6], [7]. Many fungi also have melanin in their cell walls. More subtle differences exist too, such as variations in the length and type of linkages in mannans [3], [8].

## There Are Multiple Receptors for Fungal Cell Wall Components

Considering their surface exposure and universal features, it is not surprising that components of the fungal cell wall are recognized by the innate immune system. The ability of animals to sense β-glucans is found in primitive invertebrates, most notably the horseshoe crab. In mammals, many receptors that recognize β-glucans have been described, including dectin-1, complement receptor 3 (CR3, which binds β-glucans at a site distinct from its complement binding site), and three members of the scavenger receptor family, CD5, CD36, and SCARF1 [8]–[10]. The relative contribution of each remains to be fully determined. Dectin-1, a transmembrane C-type lectin receptor highly expressed on myeloid cells, has specificity for β-1,3-glucans [8], [10]. Receptor engagement leads to phosphorylation by Src family kinases of a tyrosine-based activation-like motif (ITAM) located on dectin-1′s cytoplasmic tail and the initiation of Syk-and CARD9-dependent signaling cascades [10]. This results in phagocytosis, the respiratory burst, and activation of the transcription factors NF-κB and NFAT, leading to cytokine/chemokine gene induction. Recently, susceptibility to fungal infections has been associated with mutations in the genes encoding for dectin-1 and CARD9 [11], [12].

Two C-type lectin receptors, the mannose receptor (CD206, also known as the macrophage mannose receptor) and DC-SIGN (CD209), appear to be the major receptors on human myeloid cells that recognize mannans [3]. These two receptors have cytoplasmic motifs directing mannosylated antigens to the endocytic pathway of dendritic cells, where they can be processed and subsequently presented to T cells. The mannose receptor, which is expressed at high levels on alternatively activated macrophages, has no known cytoplasmic signaling motifs. Stimulation of the mannose receptor can lead to either proinflammatory or anti-inflammatory responses, depending upon the ligand and host cell studied [13]. Other receptors that recognize mannose residues include langerin and dectin-2. Dectin-2 and the mannose receptor also have affinity for α-glucans and chitin, respectively, although other chitin receptors undoubtedly exist [14], [15]. Thus, there is redundancy with regards to the number of host receptors that recognize glucans, mannans, and chitin. Moreover, some receptors recognize more than one fungal cell wall carbohydrate.

Surface components on fungi stimulate the toll-like receptors (TLRs) TLR2 and TLR4. With few exceptions, such as phospholipomannan of *Candida albicans*, the ligands responsible for stimulating the TLRs remain undefined. Some pathogenic fungi gain entry into the cell by displaying cell surface ligands for phagocytic receptors. The chitin-linked protein, BAD1, facilitates access of *Blastomyces dermatitidis* into macrophages via CR3, which triggers an anti-inflammatory program that fosters pathogen survival [16]. Heat shock protein 60, located on the cell wall of *H. capsulatum*, is recognized by CD18 on macrophages [4]. Commensal fungi express adhesions, many of which are mannoproteins, that facilitate colonization of epithelial surfaces [5].

## Opsonization Provides Further Means for Host Recognition of Fungi

Fungi are potent activators of the complement system, resulting in opsonization due to deposition of C3b and iC3b on the fungal surface and recruitment of inflammatory cells as a result of C3a and C5a generation. However, fungi are resistant to complement-mediated lysis, presumably due to their thick cell wall. Fungi can activate the classical, alternative, and lectin complement pathways. Normal human serum contains antibodies to fungal cell wall components, particularly mannans, that can initiate classical pathway activation upon binding. Such antibodies may also directly opsonize fungi for recognition by phagocytic Fc receptors (FcRs). Activation of the lectin pathway occurs when recognition of exposed mannans by mannose-binding lectin (MBL) triggers MBL-associated serine proteases. Recent work has emphasized the potential contribution of the ficolins and long pentraxin 3 (PTX3) to activation of the lectin complement pathway by fungi [17]. In most models, complement deficiency makes mice more susceptible to experimental mycoses.

## Fungal Masking of Ligands Can Influence Host Recognition and Pathogenicity

Most medically important fungi are opportunistic pathogens, generally attaining importance only when the individual is immunocompromised. Nevertheless, many fungi “mask” ligands, with the end result being reduced stimulation of innate immunity. The most extreme example is the capsule of *C. neoformans*, which completely eclipses the cell wall and imparts virulence on the fungus. In the absence of opsonization, encapsulated *C. neoformans* are not phagocytosed. The outer layer of mannans mostly mask β-glucans on *C. albicans* and other fungi, leaving only small amounts of β-glucans exposed [2]. α-glucans, present on some strains of *H. capsulatum*, form a layer that covers the β-glucans, thus preventing recognition by dectin-1 [6]. Recently, it was demonstrated that RodA, a surface hydrophobin that forms a rodlet layer on the surface of *Aspergillus fumigatus* conidia by covalent attachment to the cell wall, masks immunogenic determinants on the spores, resulting in a lack of dendritic cell and alveolar macrophage activation and maturation [18]. However, the rodlet layer is shed when conidia swell and germinate into hyphae. While hyphae are avidly recognized by phagocytes, they attain sizes that preclude phagocytosis. Indeed, essential to the pathogenicity of many fungi is their ability to undergo phase transition and display multiple morphotypes with differing surface properties.

## Synergism or Antagonism May Be Seen When Fungi Stimulate Multiple Receptors

Studying responses following stimulation of an individual receptor with its cognate ligand provides important insights into host responses and pathogenicity. However, during in vivo infection, a panoply of fungal ligands is displayed in variable concentrations, resulting in stimulation of multiple host cell receptors. Additionally, as fungi generally activate complement and may be recognized by antibody, both opsonic and non-opsonic recognition of fungi typically transpires. It is becoming increasingly clear that ligand combinations elicit complex patterns of inflammatory responses [8]. For example, cryptococcal mannoproteins are weak stimulators of cytokine responses. However, when TLR ligands are combined with the mannoproteins, then synergistic stimulation is observed [13]. In contrast, when dendritic cells are incubated with TLR ligands and β-glucans, synergistic production of TNFα is observed, but IL-12p70 is suppressed [19].

Immune sensing of unopsonized *C. albicans* monocytes/macrophages is mediated by at least three recognition systems composed of mannose receptors binding N-linked mannosyl residues, TLR4 binding O-linked mannoses, and dectin-1 recognizing β-glucans [8]. Adding to the complexity, following incubation with human serum, CR3 recognizes complement deposited on β-1,6-glucan branches and FcRs recognize bound IgG antibody [20].

In conclusion, common and distinct features are found when comparing cell walls of different fungi. A large number of host receptors sense cell wall components and deposited opsonins (Table 1), although some potential ligands may be masked from their cognate receptors. The nature of the innate and subsequent acquired immune response to fungal pathogens is profoundly influenced by which receptors are stimulated and to what extent.

**Table 1:** Examples of Fungal Cell Wall Ligands and Their Cognate Phagocytic Receptors.

	Receptors
**Ligands present on nearly all fungi**	
1,3 β-glucans	Dectin-1
	CR3 (CD11c/CD18)
	CD5
	CD36
	SCARF1
Mannans	Mannose receptor (CD206)
	DC-SIGN (CD209)
	Langerin CD207)
	Dectin-2
Chitin	Mannose receptor (CD206)
**Ligands present on only some fungi**	
α-glucans of *Pseudallescheria boydii*	Dectin-2
BAD1 of *B. dermatitidis*	CR3 (CD11c/CD18)
HSP60 of *H. capsulatum*	CD18
Phospholipomannan of *C. albicans*	TLR2
O-linked mannoses of *C. albicans*	TLR4
**Opsonic ligands**	
C3b	CR1 (CD35)
iC3b	CR3 (CD11b/CD18)
	CR4 (CD11c/CD18)
IgG	FcγRI (CD64)
	FcγRII (CD32)
	FcγRIII (CD16)

This table is not all-inclusive. In particular, other, as yet unidentified, chitin receptors and ligands for TLRs are likely to exist.
